# Decisions to Disclose Suicide Attempt History: A Qualitative Study

**DOI:** 10.3390/bs16091506

**Published:** 2026-08-27

**Authors:** Cassandra L. Hartman, Christine Swoboda, Carol A. Wygant, Arielle H. Sheftall

**Affiliations:** 1 Center for Suicide Prevention and Research, The Abigail Wexner Research Institute, Nationwide Children’s Hospital, Columbus, OH 43215, USA; carol.wygant@nationwidechildrens.org; 2 Center for the Advancement of Team Science, Analytics, and Systems Thinking in Health Services and Implementation Science Research (CATALYST), College of Medicine, The Ohio State University, Columbus, OH 43202, USA; christine.swoboda@osumc.edu; 3 Department of Family and Community Medicine, College of Medicine, The Ohio State University, Columbus, OH 43210, USA; 4 Department of Psychiatry, University of Rochester Medical Center, Rochester, NY 14642, USA; arielle_sheftall@urmc.rochester.edu

**Keywords:** parental–child communication, suicide, disclosure of suicide attempt

## Abstract

Individuals with a history of suicide attempt may face complex decisions about whether, when, and how to disclose their suicidal behavior history. However, little is known about how they navigate these decisions within family, social, and mental health care contexts. This qualitative study examined disclosure decision-making among biological mothers with a documented history of suicide attempt whose children aged 5–17 years had received behavioral health services. Structured clinical and demographic assessments were used to characterize the sample, followed by semi-structured qualitative interviews. Eleven audio-recorded interviews were included in the qualitative analysis. Mothers described disclosure decisions within the context of prior experiences of judgment and dismissal, stigma surrounding mental health and suicide, distrust of mental health care, structural and family stressors, and discomfort with vulnerability. Mothers also described conditions that facilitated disclosure, including close relationships with trust and open communication, and pro-mental health care attitudes. Findings suggest that disclosure of suicide attempt history is a context-dependent decision shaped by experiences across interpersonal, social, structural, and individual contexts. Rather than supporting disclosure as uniformly beneficial or harmful, these findings highlight the need for individualized, developmentally appropriate support for individuals considering whether, when, and how to discuss a suicide attempt history.

## 1. Introduction

Suicide is a top ten leading cause of death among all ages in the U.S. (Centers for Disease Control and Prevention & National Center for Injury Prevention and Control, 2024). Open discussions of mental health are consistently identified as an important component of suicide prevention (Sheehan et al., 2019; Frey et al., 2018; Brausch et al., 2025; Bettis et al., 2023; Sullivan et al., 2025; Hallford et al., 2023; Rodriguez et al., 2025; Mirichlis et al., 2024), but less is known regarding suicide attempt disclosure specifically.

Prior studies examining disclosure to other adults highlight barriers such as fear of causing distress and decision-making processes involving perceived risks and benefits (Sheehan et al., 2019; Frey et al., 2018), but do not address disclosure within the parent–child relationship. Parental disclosure decisions may be particularly complex because the meaning and potential consequences of disclosure likely vary across family circumstances. Factors such as the child’s developmental readiness, timing of the parent’s suicidal behavior, current family stressors, prior experiences discussing mental health, stigma, and anticipated responses may be relevant to whether, when, and how parents consider disclosure. Examining these contextual factors may help clarify the support parents need when navigating disclosure decisions without assuming that disclosure is uniformly beneficial, harmful, or appropriate across families.

The present qualitative study examined how mothers with a history of suicide attempt described decisions about disclosing their mental health and/or suicide attempt history to both their children and others, with particular attention to barriers, facilitators, and contextual influences shaping disclosure and nondisclosure.

## 2. Methods

We conducted a qualitative study to examine how biological mothers with a history of suicide attempt described decisions about disclosing their history to their children and others. Structured demographic and clinical assessments were administered to characterize participating mothers and their children, followed by semi-structured qualitative interviews focused on communication about mental health and suicide.

### 2.1. Study Participants

Participants were recruited from a major metropolitan children’s hospital. Eligible participants were biological parents with a documented history of suicide attempt who had a biological child aged 5–17 years receiving behavioral health services at the hospital within 12 months prior to study initiation.

Potentially eligible families were identified through review of pediatric patients’ electronic health records, specifically documentation within the family history section indicating a history of suicide attempt by at least one biological parent. Information regarding family history of suicide is routinely collected during the clinical intake process. Biological parent status was determined through medical record review, and study staff verified the parent’s self-reported history of suicide attempt during eligibility screening.

Medical record prescreening and initial recruitment were conducted under an IRB-approved waiver of HIPAA authorization. To protect privacy, mailed materials used neutral language, and suicide attempt history was not disclosed on envelopes. Access to the password-protected recruitment log was restricted to trained study staff and maintained on a secure server. Returned or undeliverable recruitment materials were documented in the recruitment log and physically destroyed to maintain confidentiality.

Exclusion criteria included inability to speak or read English, inability to provide informed consent, and non-biological caregiver status (e.g., adoptive parents, step-parents, foster parents, or legal guardians).

Eligible individuals were mailed flyers that contained a synopsis of the study. When more than one parent was documented as having a history of suicide attempt, only one parent was contacted for recruitment. Parents who were interested in participating were asked to call study staff to determine eligibility. Alternatively, parents could scan a QR code that directed them to a REDCap form where they gave their name, number, a phone number where they could be reached, and specify a day and/or time that they preferred for return contact.

Of 1056 potentially eligible parents contacted, 31 (2.9%) expressed interest in participating. Of those, 21 (67.7%) were screened and confirmed eligible, and 11 (52.2% of those screened; 1.0% of those initially contacted) consented to participate. Although eligibility included biological mothers and fathers, the final sample consisted of 11 biological mothers who completed a recorded study interview. Mothers received confirmation of their scheduled study appointment and a reminder call the day prior to the interview. Transportation and all costs associated with participation were covered by the research team.

### 2.2. Data Collection

Study team members conducted one-on-one interviews with participants between April 2024 and January 2025. Participants first completed structured demographic and clinical assessments used to characterize maternal and child histories of suicidal thoughts and behaviors. Maternal and child suicidal thoughts and behaviors were assessed using the Lifetime/Past Month version of the Columbia Suicide Severity Rating Scale (C-SSRS; Posner, 2007). Mothers completed the C-SSRS regarding their own experiences and completed a proxy report of their child’s experiences. Children did not independently complete the C-SSRS. The C-SSRS Lifetime/Past Month version distinguishes suicidal ideation from suicidal behavior and assesses actual, interrupted, and aborted suicide attempts, preparatory behavior, and non-suicidal self-injurious behaviors.

Structured assessments were followed by an individual semi-structured qualitative interview. The interview guide included questions concerning communication about mental health and suicide with the participant’s children and with other individuals (see Supplementary Materials).

Although the semi-structured interview guide also included questions related to programming for suicide prevention programming, these topics were outside the scope of the present analysis. Suicide prevention programming was examined in a previous publication from the same overarching study using a separate sample of participants who completed an earlier version of the interview guide (Hartman et al., 2025).

The present analysis used interviews collected with a subsequent version of the interview guide and focused specifically on mothers’ communication about mental health and suicide and their decisions regarding disclosure of their own suicide attempt history to their children and other people. The interview guide did not include questions about timing of participant disclosure to child (e.g., before child’s suicidal crisis, if applicable) or if the child’s age or developmental stage affected maternal decisions to disclose suicide attempt history.

For this analysis, participant disclosure of suicide attempt history was defined as confirming during their qualitative interview that they had explicitly discussed their suicide attempt history. General discussion of mental health, depression, psychiatric treatment, hospitalization, or suicidal thoughts did not meet the definition of ‘Disclosure of Suicide Attempt’ unless the mother explicitly communicated that she had made a suicide attempt. Disclosure status was determined using interview content. The codebook defining all themes used in this paper is shown in Table 1.

All interviews took place in private observation rooms and lasted between 1.5–2 h. Interviews were video- and audio-recorded, transcribed verbatim, and de-identified prior to analysis. Participants were provided breaks during the study visit as needed and received payment for participation. This study was approved by the hospital’s Institutional Review Board (IRB) and written informed consent was obtained from all participants.

### 2.3. Procedures for Minimizing Risks

Participation involved discussion of potentially distressing topics and associated assessment burden. Study staff were trained to monitor participants’ reactions to sensitive questions, and interviews could be paused or terminated if needed. If a participating mother disclosed current suicidal ideation or other significant psychiatric symptoms during the study visit, study staff contacted a crisis clinician who conducted a risk assessment and, as indicated, facilitated safety planning, follow-up with existing providers, or hospitalization when immediate safety could not be assured. Crisis consultations were conducted privately in the interview room with only the participant and research staff present.

Children were not directly enrolled or assessed as research participants. Information regarding child suicidal thoughts and behaviors was obtained through maternal proxy report using the Lifetime/Past Month version of the C-SSRS. All children met eligibility criteria based on receipt of behavioral health services at the hospital within the 12 months preceding study initiation. Mothers also completed the Service Assessment for Children and Adolescents (SACA; Stiffman et al., 2000) parent-report version to characterize their child’s behavioral health service utilization, including inpatient and outpatient treatment, counseling or therapy, and medication-related services. Because children were not participants and were already engaged in behavioral health services, the approved study protocol did not include a separate child-specific risk assessment or safety procedure.

### 2.4. Analysis

Eleven video- and audio-recorded interviews were available for qualitative analysis. One additional mother completed the study visit; however, the interview recording was unavailable due to equipment malfunction. Only interviews with complete recordings and transcripts were included in the qualitative analysis.

Interview transcripts were analyzed using deductive-dominant thematic analysis (Armat et al., 2018; Vaismoradi et al., 2013). Initial coding domains were informed by the semi-structured interview guide, while additional codes were developed inductively as concepts emerged from the data. One study team member conducted primary coding of all transcripts, supporting consistent application of the coding framework across interviews.

Coding proceeded iteratively. The primary coder reviewed transcripts, applied codes corresponding to interview-guide domains, and developed additional codes when concepts were not adequately captured by the initial framework. A senior qualitative researcher with expertise in qualitative methods provided analytic oversight through regular meetings with the primary coder to review coding decisions, discuss emergent codes, consider areas of overlap or ambiguity, and refine the developing thematic structure. A constant comparative approach was used to examine similarities and differences within and across interviews and to identify patterns relevant to mothers’ decisions to disclose or not disclose their suicide attempt history to their children.

Sample adequacy was evaluated through thematic saturation during analysis. Saturation was considered reached after analysis of nine interviews, when additional coding was no longer generating substantively new codes or themes relevant to the study aims. Because two additional interviews had already been collected before this determination was made, both were subsequently analyzed and incorporated into the final thematic analysis, resulting in an analytic sample of 11 interviews.

ATLAS.ti software (version 25.0.1) (ATLAS.ti Scientific Software Development GmbH, 2024) was used to organize transcripts and support coding and thematic analysis.

## 3. Results

Mothers were 31–48 years of age (Mean = 39 years) and had children aged 5–17 years (*M* = 12 years). All mothers had a history of suicide attempt prior to their child’s birth. Additional demographic details are presented in Table 2. All mothers reported discussing mental health in general with others, and most mothers had general mental health conversations with their child. Given the small sample size and reporting restriction recommendations, no presentation of data for cell sizes fewer than 10 (Tiwari et al., 2014; Centers for Disease Control and Prevention & National Center for Health Statistics, 2026), subgroup findings are presented descriptively to provide context for qualitative themes rather than to test group differences.

### 3.1. Mental Health Discussions and Suicide Attempt Disclosure

#### 3.1.1. Discussion of General Mental Health

While almost all mothers discussed having general (i.e., non-suicide-related) mental health discussions with their child (Table 3), one mother expressed how her own lack of readiness has prevented her from discussing mental health with her child:

**<INTERVIEWER>** Have you spoken with any of your children about your own mental health?

**<INTERVIEWEE>** No. Not yet. Will be. It’s comin’ in a couple weeks.

**<INTERVIEWER>** What has kept you from talking with them before now?

**<INTERVIEWEE>** Because I’m not mentally ready for that, it was not—what’s the point of talking to ‘em about it if I’m not emotionally in the right mindset myself? I don’t want to put *them* [emphasis added] in the situation if I’m not even ready to be in that situation myself.

(13)

**Table 3:** Examples of Maternal Discussions of General Mental Health With Child.

Theme	Participant Identifier	Representative Quote
General Mental Health	8	For example, [child] asks me about how bipolar depression actually affects me. And I even tell [child] it’s kind of hard to explain because at times you’re up and you’re happy and you feel good. And then that one second can drag you down to the mud. It’s—being bipolar, it’s just everything is just everywhere. It’s hard to think sometimes, too. I guess this—It sucks. (laughter) to have to deal with. But if [child] asks me stuff like that or if we’re even watching a video on mental health, we’ll talk about it.
	9	There’s been a couple of times where [child has] come to me and [child is] like, “I just don’t like the way my brain thinks. It doesn’t—I can’t think. I can’t think straight. I can’t keep focus.” And we’ve tried everything to try to figure out what makes [child] tick and what [child’s] safety spots are and things like that. [Child’s] not quite to the point of where [child] fully understands I don’t believe. It’s more of the, “I don’t feel right. Something’s not—my brain is just not working properly, and so I have to explain to [child] that it’s not that your brain isn’t working properly, your brain just works differently.
	14	[Child] tells me when [child’s] overwhelmed and a burden, and that’s I think that’s the biggest. [Child] feels [child is] a burden, and I’ve felt like that my whole life.

Note: Participant identifiers correspond to original study transcripts. Brackets indicate text altered for anonymity.

All mothers reported having non-suicide-related mental health discussions with others (Table 4). Disclosure to “others” referred to individuals external to the mother–child relationship (e.g., spouse/partner, healthcare provider, friend).

#### 3.1.2. Discussion of Suicide/Suicidal Behaviors

Discussion of suicide varied by participant. Some mothers discussed suicidal ideation in general, some mothers reported discussing their experience with suicidal ideation but not their suicide attempt, “No. He knows I’ve had thoughts. But not...” (14) while others disclosed their attempt history.

Some mothers reported disclosing their suicide attempt history to their child, so their child could use them as a resource if they experienced similar feelings.

[Child] usually—[child is] super mature, and so [child] usually has questions, but [child is] very—[child] always does want to know, “Mom, you would never try that now?” I’m like, “No, I do not feel like I need to try that now.” So that is a concern. “Would you ever leave?” “No, no, that’s not. That’s not it. I want you to know these thoughts that have been in my brain because if you ever have it, please come talk—this is—we can do something about this.

(10)

Some mothers discussed their child’s development stage and ability to understand affecting how much they disclosed about their suicide attempt history.

But I’ve always been open with [child]. I’ve never hid [child] from anything. I will say I leave bits and pieces out where [child is] not old enough to understand certain things. And I allow [child] to make [child’s] own decisions on things. But I don’t go into the gory details, but I just let [child] know, “Hey, yeah, this has happened, I’m not proud of it,” and I’ve showed [child] the scars from what I’ve done to myself and explain that just because it helps in the moment doesn’t mean that you don’t live with it, because you’re going to have that constant reminder, especially when it comes to self-harm, that yeah, you went through that. So, it’s more of the, “Let me just lay my heart out for you so you understand that you’re not alone,” type of conversation.

(9)

Other mothers expressed willingness to discuss their experiences with their child but not knowing when to have these discussions: “[Child], I have not had that discussion with [child] yet. I’m just waiting, I’m hoping I’ll know when the time is right” (7).

While some participants disclosed their suicide attempt to others (Table 5), one participant reported they have not discussed their attempt history outside of the study visit: “<INTERVIEWER> Have you talked to someone about your history of suicide attempt? <INTERVIEWEE> Just you” (18).

### 3.2. Contextual Influences on Disclosure

Collectively, themes illustrating parental disclosure decisions are embedded within relational, cultural, structural, and psychological contexts.

#### 3.2.1. Interpersonal Barriers

Many participants described fear of being judged and history of judgment by others, including family members, friends, and community members. Additionally, participants frequently described experiences in which their mental health concerns were dismissed and that they were encouraged not to talk about their problems even if they did exist. Examples of quotes are seen in Table 6.

#### 3.2.2. Cultural and Societal Influences

Stigma surrounding mental health and suicide was frequently cited as a barrier to disclosure. Various negative views toward those with mental health concerns were expressed by others, giving parents pause. Parents also described broader cultural messages and discussions with others concerning mental health care as unnecessary or excessive or the mental health problems did not exist (Table 7).

#### 3.2.3. Structural Stressors

Parents described financial instability, housing insecurity, and single-parent household stressors as shaping family communication dynamics. Economic challenges were described as limiting emotional availability and focus on mental health (Table 8).

#### 3.2.4. Psychological Barriers

When examining psychological barriers, some mothers described their own or their child’s distrust of mental health systems or discomfort with communicating vulnerability. Several mothers described feelings of guilt and shame surrounding suicidal behaviors and reluctance to engage in discussions even within close relationships or with their children (Table 9).

#### 3.2.5. Facilitators

Those who disclosed their suicide attempts with their child or other people described some facilitators to these conversations. Mothers were more likely to disclose within trusting relationships and to those who they had open communication with. Some mothers expressed that those who were very supportive of mental health care made talking about suicidal behaviors and receiving treatment feel more comfortable (Table 10).

## 4. Discussion

When examining maternal disclosure of suicide attempt history, most mothers who did disclose did so with other adults, whereas a small number of mothers disclosed to their children. The findings illustrate that maternal disclosure of suicide attempt history is context-dependent and the decision is embedded within mothers’ prior relational experiences, broader social attitudes toward mental health and suicide, family stressors, and comfort with vulnerability. Mothers described both barriers to disclosure and conditions that made conversations about mental health and suicide feel more possible. Importantly, these accounts suggest that the decision to disclose was not simply a matter of willingness to communicate, but involved judgments about relational safety, personal readiness, and the child’s perceived developmental readiness.

Mothers’ accounts also emphasized that disclosure was not necessarily a binary decision between telling and not telling. Some mothers described waiting until they believed a child was developmentally ready, modifying the amount or type of information shared, or postponing disclosure until they themselves felt emotionally prepared for the conversation. These accounts suggest that disclosure may be better understood as a process involving decisions about whether, when, and how much to disclose. Child age alone may not capture perceived readiness; mothers also considered maturity, ability to understand the information, and the anticipated emotional impact of disclosure. Future research should therefore examine developmental stage, timing, level of detail, and parental readiness as distinct dimensions of disclosure, including whether and how a child’s own mental health crisis or suicidal behaviors influence a parent’s decision to disclose, delay disclosure, or withhold their suicide attempt history.

Interpersonal experiences of judgment and dismissal emerged as central barriers to disclosure. Many mothers described fear of being judged, misunderstood, or stigmatized if they disclosed their suicidal behavior history. Others reflected on growing up in environments where mental health concerns were dismissed or not talked about, shaping their present-day communication patterns. These relational experiences may contribute to hesitation in initiating conversations with their own children, particularly when parents anticipate invalidation or emotional harm. Prior research has demonstrated that perceived negative responses to suicide-related disclosure can inhibit future help-seeking and open communication (Sheehan et al., 2019; Sullivan et al., 2025), reinforcing the importance of relational context in disclosure decisions.

Beyond individual relationships, broader cultural and societal beliefs about mental health influenced parental disclosure decisions. Mothers described persistent stigma surrounding suicide and psychiatric diagnoses, as well as cultural messages that mental health care is unnecessary or mental health problems do not exist. Such minimization may reflect generational norms or community-level beliefs that discourage emotional vulnerability. These findings align with research demonstrating stigma reduces both mental health help-seeking and openness in discussing suicidal behaviors (Bettis et al., 2023). In this context, maternal disclosure is not solely a personal decision but one embedded within prevailing cultural narratives about strength, resilience, and self-reliance.

Structural stressors—including financial instability, housing insecurity, and unstable or unsafe home environments—also shaped disclosure decisions. Mothers described living in “survival mode,” with limited emotional bandwidth to prioritize vulnerable conversations. When mothers are navigating economic hardship or instability, disclosure may feel secondary to immediate survival concerns. Consistent with prior work linking socioeconomic disadvantage to reduced mental health engagement (Ngamini Ngui et al., 2012; Finegan et al., 2018), structural instability may constrain opportunities for reflective, emotionally open dialogue within families.

At the individual level, psychological barriers such as distrust of mental health providers and reluctance to engage in emotionally vulnerable conversations, even with those they trust, were evident. Distrust of mental health systems and fears of negative consequences complicated disclosure decisions and receipt of mental health treatment. Mothers described internalized guilt surrounding prior suicide attempts and discomfort discussing their mental health history. In prior research, participants explained communication barriers including fear of unsupportive reactions or overburdening others as barriers to disclosing suicidal behavior (Sheehan et al., 2019). These psychological barriers likely interact with interpersonal and structural contexts and lived experience, reinforcing hesitation to disclose. Disclosure therefore appears to be a relational process influenced by both internal emotional states and external environmental factors.

The facilitators of disclosure were often the direct opposites of many of the barriers, including trust, open and transparent communication about mental health, and positive attitudes toward mental health care. Relationships with trusted friends, family, or providers were comfortable environments for disclosure even among those who may experience barriers toward disclosure in other relationships. Some mothers talked about having open communication styles with their children and the people they choose to surround themselves with that facilitate continuing discussions of mental health. The mothers who understood the utility of mental health care or had friends or family that were supportive of mental health care had positive attitudes about having mental health discussions. Prior research indicates that trust in mental health professionals positively influences receiving treatment, so some of these facilitators to disclosure may also serve as facilitators to receiving mental health treatment (Muscat et al., 2025).

### 4.1. Strengths and Limitations

This study should be interpreted while considering several limitations across structural, methodological, and sample-level domains. Structurally, participants were recruited from a single pediatric behavioral health setting within one geographic region, which may limit generalizability (e.g., parents not engaged in clinical services; families from different sociocultural contexts). All participants were female, limiting understanding of male perspectives and broader caregiver populations.

Methodologically, the small sample size and low response rate raise the possibility of self-selection bias, as parents more comfortable discussing mental health may have been more likely to participate. The low response rate may also reflect the sensitive nature of the study topic and recruitment based solely on medical record identification of suicide attempt history. The cross-sectional design precludes conclusions regarding directionality between parental disclosure and youth suicidal outcomes. Additionally, reliance on parent self-report may have introduced recall or social desirability bias, and youth perspectives were not directly assessed.

The developmental heterogeneity of the mothers’ children also limits interpretation. Children ranged from 5 to 17 years of age, encompassing substantially different developmental stages, yet the small qualitative sample did not permit systematic examination of disclosure experiences within narrower developmental groups. Mothers’ accounts suggested that perceived maturity and developmental readiness influenced disclosure, but these findings cannot determine how disclosure decision-making differs across childhood and adolescence.

The temporal context of disclosure also could not be fully characterized. Mothers varied considerably in the timing of their suicide attempts, and the interviews did not consistently establish the interval between a maternal suicide attempt and disclosure, the child’s age at every disclosure, or whether disclosure occurred before, during, or after a child’s own behavioral health or suicidal crisis. Because interviews captured experiences at one point in time, the study also cannot determine how disclosure decisions changed as children developed or as mothers’ and children’s mental health circumstances changed.

Child suicidal thoughts and behaviors were obtained through maternal proxy report rather than direct child assessment. Children were not participants and did not provide their own perspectives on maternal disclosure, their understanding of what was shared, or their emotional responses to these disclosure conversations. The study therefore characterizes mothers’ reasoning and perceptions but cannot establish how children experienced maternal disclosure or nondisclosure.

Despite these limitations, the study addresses an understudied aspect of communication about suicide. The in-depth qualitative interviews provided detailed contextual information about how mothers with a history of suicide attempt approached disclosure decisions. The interviews also allowed examination of disclosure within relational, social, structural, developmental, and individual contexts rather than treating disclosure as an isolated behavioral choice. These findings provide a foundation for more focused investigation of how parents decide whether, when, and how to discuss suicide attempt history with their children.

### 4.2. Future Directions

These findings highlight the persistent role of mental health stigma in limiting conversations about suicidal behaviors, even among parents with lived experience and children receiving behavioral health services. Efforts to reduce stigma within families, social networks, and healthcare systems may be critical for increasing parents’ comfort with discussing suicidal behaviors in developmentally appropriate ways. Expanding these conversations may allow for earlier identification of risk and more effective implementation of suicide prevention strategies that extend beyond the clinical setting and into the home.

Importantly, mothers who engaged in discussions about suicidal behaviors described these conversations as opportunities to strengthen trust and emotional closeness with their children. Interventions that support parents in navigating disclosure decisions may play a meaningful role in enhancing family-based prevention efforts.

## 5. Conclusions

In this qualitative study of mothers with a history of suicide attempt raising children aged 5–17 years, maternal disclosure decisions were shaped by multilevel influences rather than singular motivations. Findings indicate that disclosure is embedded within interpersonal experiences (e.g., judgment and dismissal), cultural and societal narratives (e.g., stigma and minimization), structural stressors (e.g., socioeconomic instability and environmental adversity), and psychological processes (e.g., shame, distrust, and reluctance).

These findings do not establish disclosure as inherently beneficial or harmful. Rather, they suggest that disclosure decisions occur within changing developmental, relational, and familial contexts and may require consideration of the needs of both the discloser and the one receiving the disclosure. Parents who described open conversations often emphasized relational trust and emotional closeness, suggesting that developmentally appropriate disclosure may serve as one pathway toward strengthening family-level protective factors.

Future research incorporating youth perspectives, more developmentally focused samples, and longitudinal designs is needed to better understand how disclosure evolves over time, how a child’s own behavioral health crisis may shape these decisions, and how disclosure is experienced by both mothers and children. Greater understanding of these processes may inform individualized, developmentally appropriate support for families navigating conversations about parental suicide attempt history.

## Figures and Tables

**Table 1:** Qualitative codebook.

Code	Definition
SA discussion—kids	When a mother explicitly discusses their suicide attempt/behavior with their children
SA discussion—other	When a mother discusses their suicide attempt/behavior with others in their life—not their children
Other mental health discussion—kids	When a mother discusses mental health with children—things other than suicidal behaviors (but does include depression, anxiety, PTSD, and suicidal ideation)
Other mental health discussion—other	When a mother discusses mental health with others—not their children, and things other than suicidal behaviors
Judgment	The mother mentions feeling judged or people being directly judgmental about mental health
Dismissal	The mother talks about themselves or others having perceptions that you should not talk about mental health issues because it is thought of as inappropriate to do so
Stigma	The mother talks about mental health stigma in society or by other people or previous generations
Minimization of mental health needs	The mother describing perceptions, their own or those of others, that people do not need mental health care or that mental health problems do not exist
Home environmental stressors	The mother talks about themselves or others having a bad home life or living environment that affects mental health
Distrust	The mother mentions personally distrusting people or systems
Communication discomfort	The mother talks about themselves or their child being “closed off” or non-communicative, independent of the reason they behave this way
Trust	The mother describes a relationship as trusting
Open communication	The mother describes people communicating openly and transparently
Pro-mental health stance	The mother talks about themselves or other people in their life being supportive of mental health care

**Table 2:** Participant Demographics.

		Parents	Children
Sex ^1^		%	%
	Female	100%	36%
	Male	0%	64%
Race			
	Asian	0%	9%
	Black/African American	27%	36%
	Multiracial	9%	0%
	White	55%	55%
	Other	9%	0%
Ethnicity			
	Non-Hispanic	91%	100%
	Prefer Not to Say	9%	0%
Household Income			
	$15,001–$30,000	18%	-
	$30,001–$50,000	36%	-
	$50,001–$75,000	9%	-
	$75,001 or More	36%	-

^1^ Sex listed on birth certificate.

**Table 4:** Example Quotes of Discussions of General Mental Health with Others.

Theme	Participant Identifier	Representative Quote
General Mental Health	7	didn’t—they don’t know about my mental health history or anything, but I—they know I have depression because I’ve admitted that.
	8	That’s (pauses). When he gets off of work, we’ll have our time when we speak, when we talk and he asks me how I’m feeling. If it wasn’t for him—he’s my rock... He’s the one I’m able to talk to to kind of clean my mind, even though I don’t tell him everything, ‘cause I don’t want him to worry.
	10	I’m not someone who is shy to discuss anything about my mental health treatment because it’s not… it’s just health. And so, I’ve talked to lots of people about mental health.

Note: Participant identifiers correspond to original study transcripts.

**Table 5:** Example Quotes of Discussions of Suicide Attempt History with Others.

Theme	Participant Identifier	Representative Quote
Friend	11	<INTERVIEWER> Did you ever speak with someone other than a mental health professional about your history of suicide attempt? <INTERVIEWEE> Mm-hmm. <INTERVIEWER> Who was that person? <INTERVIEWEE> My friend, and I talk to my pastor. That’s about it.
Significant Other	8	<INTERVIEWER> Have you ever spoken with him or anybody else about your history of suicide attempt? <INTERVIEWEE> Yeah. He knows. Yeah. I don’t talk about it a lot because I end up like this. (sighs) <INTERVIEWER> How have those conversations gone with him? <INTERVIEWEE> He understands because he’s had a rough childhood, too.
	9	So, I’ve spoken to my husband about it. I’ve also tried to have conversations with my own mother about it, to try to have her understand that it’s not something that you can just put a Band-Aid on and it heals, that it’s always going to be with me, it’s been with me for years.

Note: Participant identifiers correspond to original study transcripts. Brackets indicate text altered for anonymity.

**Table 6:** Examples of Maternal Quotes for Interpersonal Barriers.

Theme	Participant Identifier	Quote
Judgment	13	No, because I was scared. I don’t want to be judged. I don’t like to be judged because all my life, that’s all I’ve been, was judged.
	11	I couldn’t open up because my doctors were so, to me, judgmental when I didn’t think that they needed to be judgmental.
	17	There is that overhanging worry of judgment, of, “If I talk about this, am I going to have police involved?” Or, “Am I going to be hospitalized involuntarily?” That’s stuff’s traumatic. I don’t want to be othered or put my livelihood at risk because I’m being honest. And so it’s—a lot of that kind of stuff gets in the way of wanting to talk about it.
Dismissal	7	[A]cknowledging it and talking about it are just—I think those are the two biggest things that I didn’t have when I was a teenager. It was the shoved under the rug thing or nobody thought I was serious.
	14	I grew up (pause) My mom was always the, “You don’t talk about the problem,” “Just get over it,” “You’re just being dramatic,” that’s all--when I really was struggling and looking for help.
	12	My dad is a total denier to this day. So I would never be able to talk to him about anything. You can’t bring up anything to him, you just have to act like the world’s all perfect and life is perfect and fine and dandy.
	17	It’s not a widely talked about thing within our community. We don’t have any programs that we’ve even heard of.

Note: Participant identifiers correspond to original study transcripts.

**Table 7:** Examples of Maternal Quotes for Cultural and Societal Influences.

Theme	Participant Identifier	Representative Quote
Stigma	11	
	17	They still carry quite a lot of stigma. Even though they’ve been around me and my brother with our struggles, there are still things that are OK to talk about and still things that they really do not want to approach and do not--they still—they—most of them still think suicide is selfish and say that all the time. They make it very clear.
	16	Well, my parents, they probably have a diagnosable issue, but would they ever go anywhere? No. So for them, they don’t view it as a positive to take your medications because you should just be able to function. What do you need a therapist for? They’re very… (trails off). I guess it’s OK for other people, but not for their family members.
Minimization of Mental Health Needs	11	I got, ‘Ain’t nothing wrong with my kids…They just need to get the XXX over it’…As we grew up, kids didn’t have feelins.
	13	But [girl in store] was just like “Well, black people don’t really need it…we’re strong… It’s the white people that need it.”
	18	I mean, my friend is an alcoholic, so she kind of goes AA meetings and stuff like that, but I don’t think that she would ever see a psychia--I just feel like adults don’t really see the need in it because we did it as kids and it didn’t really help. So we don’t have a lot of faith in it as adults.”

Note: Participant identifiers correspond to original study transcripts.

**Table 8:** Example of Maternal Quotes for Structural Stressors.

Theme	Participant Identifier	Representative Quote
Home Environment Stressors	11	
	18	We’ve been through so much. We literally live in the same room like…And when we were homeless, we both slept on the couch.
	17	I was still living in that extremely stressful environment and so, so much of my energy was not really on me getting better … it was just very much stuck in survival mode.

Note: Participant identifiers correspond to original study transcripts.

**Table 9:** Example of Maternal Quotes for Psychological Barriers.

Theme	Participant Identifier	Representative Quote
Distrust	11	My experience with mental health has been rough and long is what I could say. I just, I don’t really like opening up about certain things to certain people.
	9	It’s so hard to have that conversation with a doctor because you’re like, “OK, if I bring this up, is—am I going to get reported?” I don’t want anything bad to happen to my children and I don’t want to go to the doctor and not trust the doctor that I’m going to and they’re going to think that I’m a bad parent because I’m saying, “Hey, I’ve got behavioral issues,” and there’s that stigma that’s stuck on it.
	18	As kids, we feel like adults are supposed to protect us. So, that kind of changed [child’s] perspective on adults. It’s a really bad feeling when no one—you don’t feel protected by anyone, you can’t trust anyone.
Communication Discomfort	8	I haven’t told [child] about my suicide attempts, because in my mind, he’s a kid, and like I said, I don’t want to traumatize my kid.
	17	with a lot of folks because it’s like, I don’t want them to feel badly.
	18	[Child] doesn’t want to talk anymore… [Child] got [it] figured out. Long as [child is] talking to somebody, I’m cool, but I’ll say, sometimes it’s better I don’t know.
	16	Maybe, looking backwards, maybe I should have spoken more openly about my experience, because it just seems kind of weird. I never told them I would cut into myself, but yet, they do the same thing.

Note: Participant identifiers correspond to original study transcripts. Brackets indicate text altered for anonymity.

**Table 10:** Example of Maternal Quotes for Facilitators.

Theme	Participant Identifier	Representative Quotes
Trust	12	I think that it’s the way [counselor] talked to me. She didn’t talk me like a doctor. She talked to me more like a friend to build that trust. And so I didn’t feel like I was paying someone to listen to my problems. So I think that’s the biggest difference is she didn’t talk to me like a patient.
	17	Well, most recently when I was in the hospital in November, I called up my best friend to let her know where I was. And she was like, “Yeah, I was anticipating your call because I got a call from a psychiatrist today saying that you had taken some action,” and she was like, “What happened?” So, she brought it before I was able to, but she also has had some serious struggles in her life, and so she is my safe person. We’ve known each other for like 12 or 13 years and she is quite literally my emergency contact.
	11	I talk to my bestie a lot. She keeps me grounded. She always tells me—I call her the yin to my yang—but she’s so—she knows where to go with me, and that’s my go-to person, not even my own sister.
Open Communication	10	And so I think just, now, people that I surround myself with are very open about it, open about mental health, nothing—I mean, I even, I was on a girls weekend last weekend for a friend’s birthday, and my one girlfriend, who I know has clinical anxiety, I mean, she was just sitting there, talking openly about it. I mean, “I’ve got my anxiety meds in my carry-on in case they lose my luggage so that I can actually have fun with you all this weekend. So, it’s like, I have chosen to surround myself with people who… you know.
	9	I talk openly with my children to let them know that it’s OK if we have these feelings, but we need to talk about it, and that way we don’t bottle it up and it blows over. So, I’ve been very, very honest, especially with my oldest, who’s now in middle school, and kind of going through [child’s] own hormone changes and everything else that’s going on. And then you have technology in the mix. So, they have everything at their fingertips. So that’s—I’m in a true state of just having that open and honest conversation and letting my kids know that, “Hey, it’s—you’re not weird.” It’s not—it’s nothing to be shameful about. Everyone has thoughts in some capacity of, “I don’t want to be here anymore, I can’t handle this, I can’t take it.”
	10	I have always been very open with [child]—when [child] got to a certain age—because I always wanted [child]—like I said, I shared with you that when I’ve had my own things happening as I’m growing up, I didn’t feel there was maybe enough done, maybe that could have impacted something in my life along the way. I don’t know, but I always wanted [child] to feel like [child’s]—not even just me, but [child’s] dad—that we were a safe place and that [child] could always talk to us about anything.
Pro-Mental Health	7	But my experience with counseling is just airing the feelings, having someone validate it, and/or contradict what you’re feeling and saying with reality. Helping you just to—helping me to see a different way…and get a better grasp and say, you know I hadn’t thought of it that way and that’s good, you know. Definitely healthy, I’m all for it. I think everyone should get it.
	10	<INTERVIEWER> Yeah. And can you describe how your family and friends view treatment for suicide and suicidal behaviors? <INTERVIEWEE> Again, in a positive light, I mean this is something that’s just taking care of another health issue.
	12	Or we just, we’re very open about mental health because it’s important to me that we destigmatize it so that [child] gets the care that [child] needs when [child] needs it.

Note: Participant identifiers correspond to original study transcripts. Brackets indicate text altered for anonymity.

## Data Availability

The raw data supporting the conclusions of this article will be made available by the authors on request.

## Supplementary Materials

The following supporting information can be downloaded at: https://www.mdpi.com/article/10.3390/bs16091506/s1, Interview Guide for Parents with a History of Suicide Attempt.

## Abbreviations

The following abbreviations are used in this manuscript:

CDC	Center for Disease Control and Prevention
STB	Suicidal Thoughts and Behaviors
C-SSRS	Columbia-Suicide Severity Rating Scale
