# Supplementary material for: Decisions to Disclose Suicide Attempt History: A Qualitative Study
Source: Behav Sci (Basel). 2026 Aug 27;16(9):1506. doi: 10.3390/bs16091506 (PMC13603723; doi:10.3390/bs16091506)
Supplement: Supplementary file 1 [file behavsci-16-01506-s001.zip › behavsci-4307820-supplementary.pdf]

Interview Guide for Parents with a History of Suicide Attempt

Thank you for agreeing to speak with me today. We are interested in learning from you about your and your child's experiences with mental health services. Specifically, we will be asking questions about how you discuss mental health and getting your opinion on suicide prevention programming. Please know that **you do not have to answer any question you do not wish to answer. If you don't want to answer a question, you can just say "skip" or "pass" when a question is asked that you don't want to answer.**

Section1: Parent's Mental Health Treatment

I would first like to learn from you about your own experiences with mental health service such as treatment, or support of some other kind like programs through the community.

- Can you describe your experiences?
- Can you share about a time that you spoke with another person about your history of mental health services??
    - Did you ever speak with someone besides a mental health professional about your history of suicide attempt? Who was that person? Can you tell me how that conversation started and how it went?
- Have you spoken with your child about your own mental health, mental health treatment experiences, and/or history of suicide attempt?
    - If yes, how do you talk to your child about this topic? Are you comfortable sharing about the kinds of things you say to your child about this? How do you feel about these conversations? How does your child respond to these conversations?
- How have your experiences with mental health services and mental health professionals impacted in your decisions related to seeking services for your child?

Section 2: Child's Mental Health Treatment

Now I am going to ask some questions about your child and their experiences with mental health treatment.

- Can you share about a time that your child spoke with you about their mental health?
- What led to you getting your child mental health services?
    - What specific behaviors or needs were you hoping to address?
    - Could you describe any changes in behavior or important events that happened in your child's life prior to you seeking mental health services for your child?
    - Would you be willing to share a story about a time that your child was doing the concerning behavior(s)?

- How has your child's behavior changed since starting their mental health treatment?
- Can you describe how you are involved in your child's mental health treatment?
    - Are there things you would change about your involvement in your child's mental health care?
        - If yes, what would those changes be and look like?
          If no, why is that so?

Section 3: Perspectives about Suicide Prevention Programs
I am now going to ask you to think about suicide prevention programs and how they might look for youth with a parental history of suicide attempt.

- Can you tell me thoughts about suicide preventions specific for this population of youth?
- If a suicide prevention program was created specifically for youth with a parental history of suicide attempt, what do you think the prevention program would look like?
    - What would be the key program features?
- At what age do you think suicide prevention efforts should begin? Why?
    - Do you have ideas about what, specifically, might be helpful to include for a suicide prevention program for that age?
- In what type of setting or location do you think suicide prevention programs would work best? Why?
- Ideally, who would provide these kinds of prevention programs?
- What do you think would make it easier for families or youth to participate in a program like this?
- Do you think parents should be involved in this kind of prevention program?
- Can you describe any barriers that you think might make it difficult for families or youth to engage in a program like this?
- What would make the prevention programming appealing?
- If your child were not showing behaviors that are concerning, how likely would you be to to seek suicide prevention programming?
- How would you feel if your child attended an intervention/program to prevent suicide?

Section 4: Stigma, Mental Health and Suicidal Behavior
I am now going to ask you how your friends, family, and community view mental health and suicide/suicidal behavior.

- When thinking about your family and friends, can you describe how they view suicide/suicidal behavior?
- Can you describe how people in your community view suicide/suicidal behavior?
- Can you describe for me how people in your community view treatment for suicide/suicidal behaviors?

- Can you describe for me how people in your community view suicide prevention programs?

<u>Section 5: Final Thoughts</u>

- Is there anything else you'd like to tell us about suicide prevention for youth that you think we should know?

Thank you so much for your time and participation! Your responses were very helpful.
